# Conversion of Chitin to Defined Chitosan Oligomers: Current Status and Future Prospects

**DOI:** 10.3390/md17080452

**Published:** 2019-08-01

**Authors:** Christian Schmitz, Lilian González Auza, David Koberidze, Stefan Rasche, Rainer Fischer, Luisa Bortesi

**Affiliations:** 1 Aachen-Maastricht Institute for Biobased Materials, Maastricht University, Brightlands Chemelot Campus, Urmonderbaan 22, 6167 RD Geleen, The Netherlands; 2 Department Plant Biotechnology, Fraunhofer Institute for Molecular Biology and Applied Ecology IME, Forckenbeckstraße 6, 52074 Aachen, Germany; 3 Indiana Bioscience Research Institute, 1345 W 16th St #300, Indianapolis, IN 46202, USA

**Keywords:** chitin, chitosan, chitosan oligomers, deacetylation, depolymerization, enzymatic conversion, design of experiments

## Abstract

Chitin is an abundant polysaccharide primarily produced as an industrial waste stream during the processing of crustaceans. Despite the limited applications of chitin, there is interest from the medical, agrochemical, food and cosmetic industries because it can be converted into chitosan and partially acetylated chitosan oligomers (COS). These molecules have various useful properties, including antimicrobial and anti-inflammatory activities. The chemical production of COS is environmentally hazardous and it is difficult to control the degree of polymerization and acetylation. These issues can be addressed by using specific enzymes, particularly chitinases, chitosanases and chitin deacetylases, which yield better-defined chitosan and COS mixtures. In this review, we summarize recent chemical and enzymatic approaches for the production of chitosan and COS. We also discuss a design-of-experiments approach for process optimization that could help to enhance enzymatic processes in terms of product yield and product characteristics. This may allow the production of novel COS structures with unique functional properties to further expand the applications of these diverse bioactive molecules.

## 1. Introduction—Chitin, Chitosan and Chitosan Oligomers

**Figure 1:**
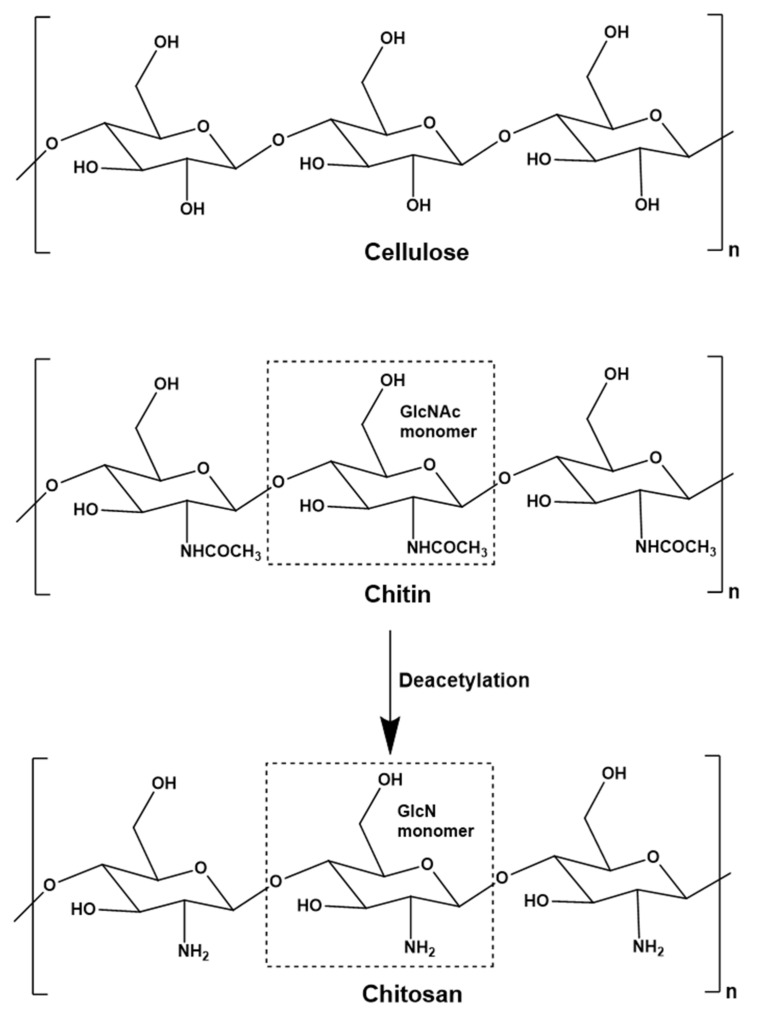
Chemical structures of cellulose, chitin and its fully deacetylated derivative chitosan. Whereas cellulose is a polymer of glucose, *N*-acetylglucosamine (GlcNAc) and glucosamine (GlcN) are the monomeric constituents of the chitinous polysaccharides.

Chitin is a natural polysaccharide predominantly found in the cell walls of fungi and the exoskeletons of crustaceans and insects. It is the second most abundant polysaccharide in the world after cellulose, and the global annual turnover is ~10^11^ tonnes [1]. The seafood industry generates ~10^6^ tonnes of chitin annually as a waste stream, much of which is composted or converted to low-value products such as fertilizers, pet foods and fishmeal [2]. The chemical structure of chitin (*N*-acetyl-1,4-β-D-glucopyranosamine) is similar to that of cellulose due to the β(1-4) linkage of the monomers – these are glucose in cellulose and predominantly *N*-acetylglucosamine (GlcNAc) in chitin (Figure 1). Although chitin is generally defined as a polymer of GlcNAc, natural chitin is actually a co-polymer of randomly distributed glucosamine (GlcN) and GlcNAc units, with the latter much more abundant. Three allomorphs of chitin occur naturally, differing in terms of microfibril orientation: α-chitin, β-chitin and γ-chitin [3,4,5]. Chitin is tough, inelastic and insoluble in aqueous media, reflecting the abundance of hydrogen bonds that form between acetamido groups in adjacent polymer chains. Chitin therefore has few industrial applications, although it can be used as a matrix material for the purification of enzymes with carbohydrate-binding domains [6,7,8] and as membrane material for the retention of proteins [9,10]. Additionally, smaller chitin oligomers are used in the agricultural industry as plant growth stimulators [11,12].

**Figure 2:**
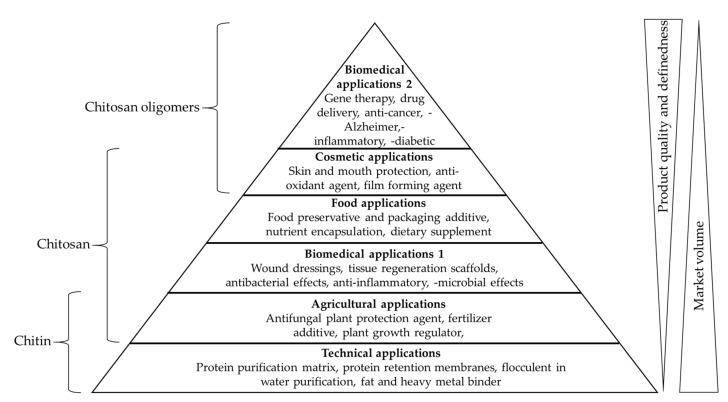
Applications of chitin, chitosan and chitosan oligomers (COS) ordered according to their application fields, market volume and product quality. Biomedical applications are split into two segments: applications for bulk chitosan and applications for more defined COS [14,25,31].

Chitin is the primary feedstock for the production of the commercially more relevant polymer chitosan (Figure 1), as well as chitosan oligomers (COS). Like chitin, chitosan is a co-polymer of GlcN and GlcNAc units, but GlcN is the predominant constituent [3]. Chitosan is therefore considered a deacetylated derivative of chitin, often defined by the degree of acetylation (DA). Chitosan becomes chitin when the DA reaches 50%, but for practical reasons the name chitosan continues to be used for molecules with a lower DA, and chitin is typically reserved to describe polymers where the DA is ~100%. In addition to the DA, the properties of chitin and chitosan also depend on the degree of polymerization (DP), which is the chain length and correlates with the molecular weight (MW) in kDa [13]. The DP is a useful term when the chain length is precisely defined, for example DP6 is a hexamer. For larger oligomers and polymers, the chain length tends to vary within a certain range, and the DP may be given as an approximate or average value, or the average MW in kDa may be used instead. Unlike chitin, chitosan is soluble in acidic aqueous solutions. Chitosan can therefore interact with other molecules in solution and shows wide-ranging biological effects, including antibacterial, antifungal, anti-inflammatory and anti-cancer activities, as well as fat-binding, film-forming, antioxidant and chelating capacities, leading to applications in several industry sectors [14]. In the food industry, chitosan is used as a preservative, a packaging additive, a dietary supplement, and as a nutrient encapsulation system [15]. In the cosmetic industry, chitosan is used as an antioxidant and antibacterial agent in skin protection products, toothpaste and mouthwash, and as a film-forming agent in shampoos and lotions [16]. In the agricultural industry, chitosan is used to protect plants from bacteria, fungi and viruses, as a plant growth regulator, and as a fertilizer additive [17,18,19,20]. In the wastewater treatment industry, the fat-binding and chelating properties of chitosan are exploited for the removal of fats, dyes and heavy metals [21,22,23,24]. Chitosan is biocompatible, biodegradable and non-toxic, so its antimicrobial and anti-inflammatory properties are also ideal for medical applications [25,26,27]. Chitosan is therefore used as an antibacterial agent in wound dressings, as a non-viral vector for gene therapy, and chitosan hydrogels are used as drug delivery systems and for the treatment of cancer [28,29,30]. Finally, chitosan-based scaffolds are used in tissue regeneration [31] (Figure 2). 

The medical and technical applications of chitosan are however restricted because of its low solubility at physiological pH, but COS are more soluble and are therefore more suitable for medical and cosmetic applications [32]. COS also possess bioactive properties lacking in the longer chitosan polymer because these small molecules can penetrate cell membranes, allowing them to influence gene expression and biological processes such as apoptosis [33,34]. The medical applications of COS therefore combine the antimicrobial, anti-inflammatory and antioxidant properties of chitosan with enhanced solubility and the ability to specifically bind nucleic acids and certain drugs [35,36,37,38,39,40,41,42]. Recent studies have revealed a strong structure–function relationship for chitosan and COS [13,43,44]. The DP and DA determine general properties such as solubility and mode of chemical interactions, but another important property is the pattern of acetylation (PA), which can be defined as the sequence of GlcN and GlcNAc units along the molecular backbone [45]. Chitosan with a higher molecular weight (28–1671 kDa) has stronger antimicrobial effects than COS with a lower molecular weight (1–22 kDa) [13]. However, the antimicrobial activity of chitosan and COS reflect three different modes of action dependent on the DP and DA: (1) interaction with molecules on the cell surface, (2) interaction with the cell membrane, and (3) interactions with intracellular targets [46]. COS within the DP range 2–15 show higher antifungal activity than chitosan (MW 2000 kDa) [47]. The anti-tumor effects of COS are 100% higher when using short and fully deacetylated chains (DP1–DP8, DA = 0%) rather than chitosan (MW ~1900 kDa, DA = 1.5%) [33]. The PA also has a significant effect on biological activity [48]. These findings indicate that: (1) the reliable production of chitosan/COS with defined properties is necessary to ensure consistent biological and physiological activity and (2) COS are more soluble and in some cases more physiologically active than chitosan. Given the infinite variety of molecules that could be produced by systematically controlling the DA, DP and PA, the production of COS that do not occur in nature could allow the discovery of new mechanisms of action and potentially new biological and physiological functions.

## 2. Chitin Extraction from Marine Biowaste

**Figure 3:**
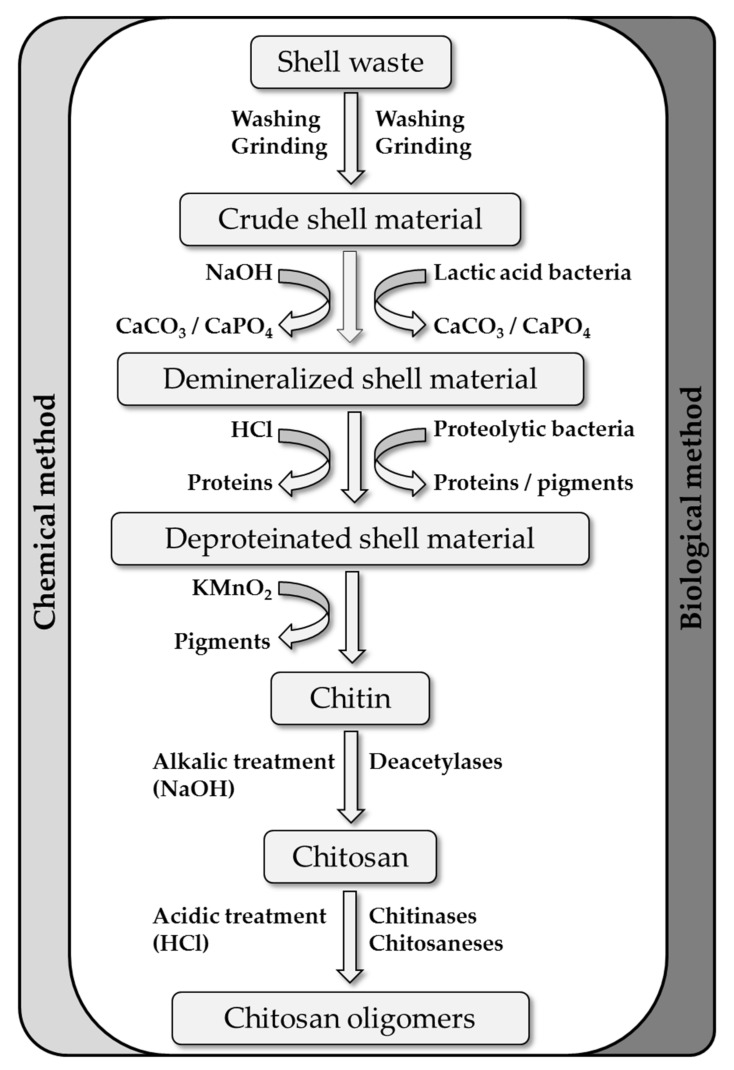
Chemical and biological methods for the extraction of chitin and its sequential conversion to chitosan and chitosan oligomers.

Chitin is mainly found in fungal cell walls and in the exoskeletons of crustaceans and insects, but also in some algae and micro-algae. Although crustacean shells are the most abundant and easily accessible source of chitin [1], the other sources yield α-chitin and β-chitin of either similar or more consistent quality than crustaceans [49,50,51,52,53,54,55,56]. Furthermore, algal sources of chitin are gaining attention in the cosmetic and food industries as animal-free/vegan products [57,58,59]. Most chitin/chitosan is nevertheless derived from crustacean shell biomass, which can be considered a natural composite material comprising 30–40% structural proteins, 30–50% CaCO_3_ and various forms of calcium phosphate, and 20–30% chitin [60] along with smaller quantities of pigments and lipids [61]. The processing and valorization of this material begins with the extraction of chitin, and this involves washing and grinding, the two major steps of demineralization and deproteination [62], and the removal of pigments and lipids using KMnO_2_. Demineralization or decalcification typically involves treatment with HCl or other acids such as HNO_3_, H_2_SO_4_, CH_3_COOH or HCOOH to produce soluble calcium salt [61,62,63,64]. The deproteination of shrimp waste for industrial applications is usually achieved by mixing with NaOH at temperatures up to 160 °C, but other alkalis such as KOH, Na_2_CO_3_, NaHCO_3_, K_2_CO_3_, Ca(OH)_2_, Na_2_S, CaHSO_3_ and Na_3_PO_4_ have also been used successfully [61,65,66,67]. However, these treatments also cause the random cleavage of the chitin backbone and random deacetylation, resulting in chitosans and COS that are undefined in terms of DP and DA [61,65,67,68]. Biological methods can reduce the environmental burden of the alkali/acid steps and also avoid unwanted changes to the chitin structure [69]. In such methods, naturally produced lactic acid is used for demineralization and proteases are used for deproteination [70,71,72]. Additionally, whole bacterial extraction procedures have been developed involving the co-fermentation of proteolytic and lactic acid bacteria on shrimp waste, resulting in milder treatment conditions and a better-defined chitin product [69,73,74,75] (Figure 3).

## 3. Chemical Conversion of Chitin to COS

### 3.1. Chemical Deacetylation of Chitin

The chemical production of COS is usually initiated by the deacetylation of insoluble chitin raw material. This increases the solubility of the substrate for the subsequent depolymerization step, which yields water-soluble COS molecules. Because the biological functions of COS depend on the DA, further limited deacetylation is carried out to generate oligomers with a DA in the range 0–50%. The chemical removal of acetyl groups from chitin can be achieved either by acid or alkali hydrolysis. However, acidic deacetylation also causes the cleavage of the chitin backbone thus reducing the DP. Therefore, hot alkaline deacetylation (>80 °C) is the method of choice for the production of chitosan and COS because depolymerization is less prevalent [76]. The most common reagent is NaOH, and the DA is determined by the NaOH concentration (25–50%), reaction temperature (80–120 °C) and reaction time (1–24 h). However, prolonged high-temperature alkali treatment also causes depolymerization [77]. An extensive investigation of the abovementioned process parameters as well as the effect of additives (NaBH_4_ and thiophenol) revealed that the high-molecular-weight (50–1000 kDa) chitosan can be produced with a well-controlled DA by using fixed reaction conditions (50% NaOH, 120 °C in the presence of NaBH_4_) and varying the reaction time between 3 and 12 h [64,78]. Similarly, chitin processing conditions have been screened systematically (25% or 50% NaOH, for 2, 5 or 10 h, 100 °C) revealing that the higher NaOH concentration significantly increased the reaction rate and prolonged incubation favored the recovery of low-molecular-weight chitosan [62]. In another investigation, chitin was incubated at 80 °C or 90 °C in 45% NaOH for 20, 60 and 120 min with similar results [79]. Chitosans with DA values of 21–29% and 15.6% have been produced by varying the NaOH concentration, temperature and time in a screening experiment, revealing that all three parameters must be high (120 °C, 24 h, 40% (*w*/*v*) NaOH) to reduce the DA, but this also resulted in a significant reduction in the DP [77]. Statistical experimental designs using response surface methodology (RSM) have also been used to find optimum conditions for efficient deacetylation while avoiding severe depolymerization. For example, one study revealed interdependencies between the incubation time, temperature and NaOH concentration and showed a synergistic effect in which the DA (4.3–32.7%) and DP (100–1100 kDa) both decreased as the temperature, reaction time and NaOH concentration increased [80]. RSM has also been used to optimize the deacetylation/depolymerization relationship, revealing that a temperature of 130 °C and a reaction time of 90 min allowed the production of COS with an average MW of 150 kDa and a DA of 10% [81]. Based on these empirical optimization studies, alkaline deacetylation conditions can now be selected to produce chitosan within a desired DA range of 0–50%. However, the harsh conditions inevitably have a simultaneous effect on the DP, which makes it difficult to produce specific COS using chemical methods. It would therefore be desirable to decouple the deacetylation and depolymerization reactions. Chemical deacetylation methods are also nonspecific so it is not currently possible to control the PA. 

### 3.2. Chemical Depolymerization of Chitosan

The conversion of chitin/chitosan into shorter oligomers requires the hydrolysis of the glyosidic bond between the GlcNAc and/or GlcN units. Various approaches for chemical depolymerization have been tested using HNO_2_, HF and H_2_O_2_ [82,83], but acid hydrolysis with HCl remains the most common and effective method [35,43,45,84]. However, chemical hydrolysis achieves low yields of COS and favors the production of monomeric GlcNAc/GlcN. Additionally, COS prepared by acid hydrolysis are usually excluded from human medical applications because the toxic hydrolysis reagents cannot be removed completely [85]. A defined and consistent DP can only be achieved by the assessment and control of physicochemical factors that influence the depolymerization process. HCl has selective activity according to the specific nature of the glycosidic bond in the chitin/chitosan backbone, with threefold higher activity on GlcNAc–GlcNAc and GlcNAc–GlcN bonds compared to GlcN–GlcN bonds [86]. However, given the random distribution of GlcNAc and GlcN in natural chitinous polymers, this selectivity cannot be exploited to achieve a specific DP. Therefore, the DP has been optimized by manipulating various physicochemical process parameters, as discussed below. A mixture of chitin and chitosan oligomers ranging from dimers (DP2) to dodecamers (DP12), was prepared by the acid hydrolysis of fully deacetylated chitosan (DA = 0%) using 12 M HCl followed by differential precipitation to first remove larger oligomers using NaOH and sequentially adding ethanol to precipitate the desired smaller oligomers. Acetic anhydride was then added in different amounts to partially re-acetylate the COS, achieving specific DA values of 25%, 40%, 60%, 80% and 90% [82]. A similar selective fractionation approach using methanol (70%, 80% and 90%) was used to enrich for COS with a DP > 6 following the acid hydrolysis of chitosan [87]. The inclusion of small quantities of H_2_O_2_ in the acid hydrolysis mix yielded COS mixtures of DP ≤ 9, and increasing the H_2_O_2_ content (1–5%), reaction time (0–6 h) and temperature (60–100 °C) led to higher reaction rates, product yields and a broader COS distribution in terms of DP [83]. Acidic hydrolysis has also been carried out using acetic acid spiked with H_2_O_2_ to partially degrade the chitosan, followed by ultrafiltration to isolate COS with different DP ranges [32]. The correlation between the H_2_O_2_ concentration and incubation time has been investigated with or without microwave radiation, revealing conditions that allow the production of COS with a better-defined MW, but it was not possible to produce COS with a MW below 2000 Da using this microwave enhanced degradation procedure [43]. Hydrolysis of chitosan in the presence of diluted HCl (1.8 M) and three different zeolite adsorbents (HZSM-5, molecular sieves beads 0.3 nm and 1.0 nm) was also carried out and yielded COS up to DP 9. Furthermore with this method it could be shown that higher yields can be achieved as the hydrolysis equilibrium is shifted more towards the products [88]. Finally, chitin tetramers, pentamers and hexamers have been produced by the acid hydrolysis of chitin with concentrated HCl at 40 °C followed by a sequential differential acetone precipitation method [89]. The chemical hydrolysis of chitin can rapidly yield mixtures of COS. However, inorganic catalysts such as HCl and H_2_O_2_ do not achieve sufficient intrinsic substrate specificity and reaction selectivity to effectively produce COS with a defined DP. Laborious additional purification approaches are therefore required such as preparative gel permeation chromatography and cation exchange chromatography to separate COS into size-specific fractions [84]. Chemical hydrolysis also affects the DA and there is no way to control the PA. These issues can be addressed by replacing chemical hydrolysis with biological methods for the preparation of COS, specifically the use of chitinolytic enzymes such as chitinases, chitosanases and chitin deacetylases. 

## 4. Biological Conversion of Chitin to COS

### 4.1. Enzymatic Deacetylation of Chitin

**Figure 4:**
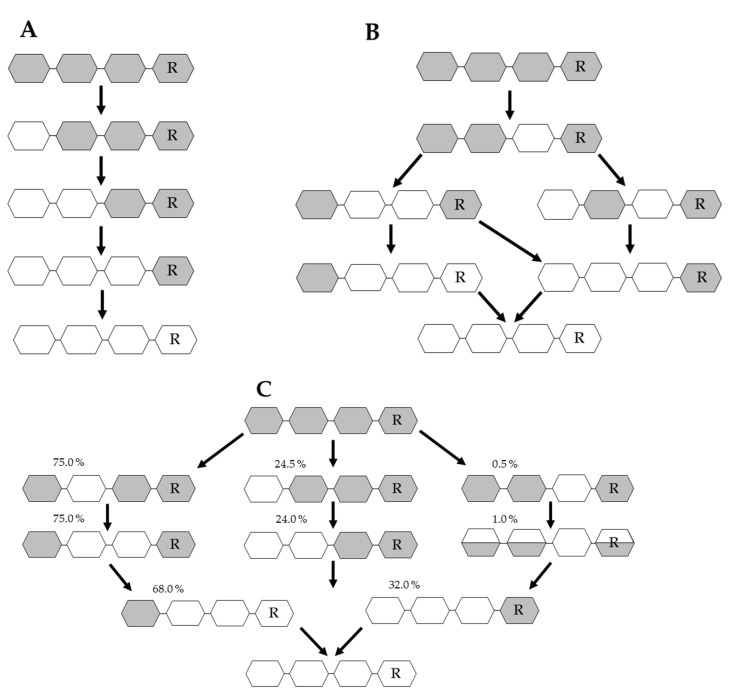
Overview of different pathways of the enzymatic deacetylation of chitin oligomers. (**A**): Deacetylation of a chitin tetramer by *M. rouxii* CDA in a ‘multiple attack mode’. (**B**): A chitin tetramer that is deacetylated by a *C. lindemuthianum* CDA. This is done in a ‘multiple chain mode’. (**C**): Subside capping deacetylation model used to explain the generation of partially deacetylated COS using a recombinant CDA from *Puccinia graminis f. sp. Tritici.* R: sugar with reducing end; grey box: GlcNAc; white box: GlcN; %: relative conversion yield; halved box: three different variants can be generated from the precursor oligomer as only one of the acetylated sugars gets deacetylated. [94,99].

Chitin deacetylases (CDAs, EC 3.5.1.41) catalyze the hydrolysis of GlcNAc in chitin and COS, to produce chitosan, partially acetylated COS, or fully deacetylated glucosamine oligomers [90]. CDAs have been identified in marine and soil bacteria, several fungi, a few insects and at least one virus. All CDAs belong to the carbohydrate esterase family 4 (CE4) and share a conserved NodB homology domain (polysaccharide deacetylase domain) which is required for their catalytic activity [91]. The first active CDA to be purified and characterized was from the fungus *Mucor rouxii* in 1970, and it has a MW of 75 kDa and an optimum pH of 5.5 [92]. Since then, CDAs have also been isolated from the fungi *Absidia coerulea, Aspergillus nidulans,* and *Colletotrichum lindemuthianum* [93,94]. Their physicochemical properties (MW, optimum pH and temperature, effect of metal ions, and substrate specificity) have been studied extensively [94]. In order to identify enzymes that produce well-defined COS and understand the reaction process, substrate specificity and catalytic mechanism, further CDAs from organisms such as archaea, marine bacteria and insects have been isolated, purified and characterized in addition to those from fungi (Table 1) [94,95]. Depending on the substrate and the reaction conditions, CDAs can produce different fully or partially deacetylated COS that are well defined in terms of DA and PA. Two fungal CDAs have been studied in particular detail, revealing distinct catalytic mechanisms known as the multiple-attack (Figure 4A) and multiple-chain (Figure 4B) mechanisms, respectively. The CDA from *M. rouxii* (Zygomycetes) binds to the polysaccharide chain and several sequential deacetylations take place [96]. This exo-type enzyme deacetylates chitin oligomers with a DP > 2, with sequential deacetylation at the non-reducing end of the oligomer, yielding D_n_ as the acetylation pattern (Figure 4A) [97]. In contrast, the CDA from *C. lindemuthianum* (Ascomycetes) uses the multiple-chain mechanism in which the enzyme forms an active enzyme–polymer complex and hydrolyzes a single acetyl group before dissociating and forming a new active complex, which binds to another chain [98]. The enzyme fully deacetylates DP3 and DP4 chitin oligomers (Figure 4B), but the reducing end (GlcNAc)_2_ cannot be deacetylated [94]. 

An additional deacetylation mechanism, the “subside capping deacetylation model”, has also been identified, in which several catalytic events take place on a single substrate molecule and lead to sequential deacetylation (Figure 4C). This mechanism is used by bacterial CDAs such as *Rhizobium* spp. NodB and *Vibrio* spp. CDA, which deacetylate a specific position leading to deacetylated products [100]. Most bacterial CDAs preferably act on low-molecular-weight COS and are essentially inactive on polymeric chitin and chitosans. Such enzymes have been found in *Vibrio* spp. and more recently in *Shewanella*, a genus of marine bacteria living in extreme aquatic habitats [101,102]. The best-known CDAs that produce COS with a defined DA are *Rhizobium* spp. NodB and VcCOD from *Vibrio choloreae* [48,91,103]. Both enzymes can produce COS with the same deacetylation pattern (ADA_n-2_) (Table 1). The enzymes have been combined in a single reaction to generate double-deacetylated COS with a novel and defined PA (DDA_(n-2)_) [48]. This was the first study describing the preparation of novel chitosan oligomers with a previously unreported PA. More recently a CDA from the soil bacterium *Arthrobacter* sp. (ArCE4A) was found to accept COS substrates with a DP ≥ 2. This enzyme uses a multiple-chain mechanism to produce different mono/di-deacetylated products with a defined PA (D_(n-1)_ A) [104]. Defined COS can also be produced using fungal CDAs. For example, the CDA from *Puccinia graminis* (PgtCDA) has been expressed in *Escherichia coli* and its regioselectivity was tested on different chitinous substrates [99]. The enzyme showed activity towards soluble glycol-chitin and partially acetylated chitosan polymers, yielding a product with a novel PA (AAD_n_, where *n* > 1) (Table 1). The expression of several recombinant fungal CDAs has been achieved in various eukaryotic hosts and in *E. coli* by codon optimization, fusion to solubility-promoting proteins and targeting to different subcellular compartments [99,105,106,107,108,109,110,111,112] (Table 1). The recent expression of a CDA from the nematophagous fungus *Pochonia chlamydosporia* (PcCDA) revealed its activity on COS with a DP > 3, generating mono/di-deacetylated products with a different PA compared to closely-related fungal CDAs [95]. PcCDA selectively converts a DP5 substrate into single mono-deacetylated products in the penultimate position from the non-reducing end (ADAAA), and then transforms them into a di-deacetylated product (ADDAA). This novel PA produced by this enzyme provides further insight into the substrate specificity of this enzyme family. Finally, 14 partially-acetylated chitosan tetramers have been produced by combining different bacterial, fungal and viral chitin deacetylases [113]. 

This study showed for the first time that a range of CDAs from different origins can acetylate COS with the same regioselectivity displayed during the conventional deacetylation reaction. Forward and reverse reactions were performed for each enzyme, with the forward reaction causing the tetramer to be partially deacetylated and the reverse reaction resulting in glucosamine re-acetylation [113]. The PA of oligomers produced by CDAs have recently been explained based on the subsite capping model, developed by comparing the structures of five CDAs in the CE4 family [99] (Figure 4). The model provides insight into the position of secondary protein-structures such as α-helices, β-sheets and loops. Furthermore, it was shown that the position of loop- structures decorating the active site are key elements in the substrate specificity of different CDAs. Understanding the structure–function relationship of enzymes in the presence of their substrate can help to explain the regioselectivity of deacetylation and the resulting PA [99,100,115]. 

In summary, a plethora of diverse CDAs have been characterized in terms of their physicochemical properties and deacetylated products. The substrate specificity and selective reaction mechanisms of CDAs can, in contrast to chemical methods, consistently generate COS with a defined DA and PA. Further studies are required to understand the structure–function relationships of partially acetylated COS, which will enable targeted applications in the food industry, agriculture and medicine.

### 4.2. Enzymatic Chitin/Chitosan Depolymerization

The biological conversion of chitin polysaccharides into shorter oligomers requires hydrolytic enzymes that contain conserved chitin-binding domains and chitin-specific active sites. Many chitinolytic enzymes are produced by plants, fungi and bacteria (Table 2). All of them are glycosyl hydrolases, but they differ in terms of reaction mechanism, thermostability and product characteristics [116]. Chitinolytic hydrolases can be categorized according to their mode of action. Endo-chitinases (EC 3.2.1.14) bind randomly to a chitin polysaccharide strand and hydrolyze internal glycosidic bonds producing various fragment sizes ranging from dimers to polymers. These enzymes are required for the production of COS because they partially reduce the DP of chitin. In contrast, exo-chitinases (EC 3.2.1.29) bind to the reducing or non-reducing end of chitin and release monomeric (DP = 1) and to lesser extent dimeric (DP = 2) GlcNAc units. These enzymes are necessary for the complete degradation of chitin, and are therefore of only secondary interest for the production of larger COS (DP > 2). Chitosanases (EC 3.2.1.132) are more selective chitinolytic enzymes because they hydrolyze GlcN-GlcN bonds and their activity is therefore dependent on the DA and PA [117]. Finally, chitobiases (EC 3.2.1.29) cleave GlcNAc dimers to release GlcNAc monomers [118]. Other enzymes such as cellulase and lysozyme are also known to exhibit some hydrolytic activity towards chitin and chitosan but are not specific for these substrates [32,119]. COS with a size range of 5–30 kDa have been produced by fractionated enzymatic hydrolysis using a mixture of a cellulase, pepsin and lysozyme [32].

Chitinases and chitosanases from various sources have been expressed as recombinant proteins in heterologous host systems such as *E. coli* and *Bacillus subtilis* [121,123,128,129,130,131]. Chitinase D from *Serratia proteamaculans* was used by to convert chitosan polymers (DA = 35% and DA = 61%) into undefined COS mixtures varying in DA and with a DP ≥ 6 after incubation for 90 min. Purified specific COS were also obtained after size exclusion chromatography for bioactivity testing [120]. RSM has been used to statistically optimize the production of a *Purpureocillium lilacinum* CFRNT12 chitosanase by solid-state fermentation and the enzyme was used to produce mixtures of different COS from colloidal chitosan [122]. Interestingly, a new class of chitosanases from the fungus *Alternaria alternata* has been identified that selectively cleaves after GlcN-GlcNAc pairs and thus shows no enzymatic activity against fully acetylated or fully deacetylated substrates, making the enzyme ideal for the production of novel structurally defined COS [128]. The secretion of a heterologous chitosanase by *B. subtilis* was optimized to generate mixtures of chitosan dimers, trimers and tetramers from chitosan [132]. Eight different chitinolytic bacteria and 20 chitinolytic fungi were isolated from soil samples, and by incubating the secretomes with chitosan polymers it was possible to produce diverse mixtures of COS (DP1–DP6) as well as larger oligomers, providing another potential source of novel COS for medical and industrial applications [127]. Recently, a GH46 family endo-chitosanase isolated from the rhizobacterium *Gynuella sunshinyii* was expressed in *E. coli* and used to produce short COS with a consistent DP when incubated with chitosan [114]. Similarly, a GH8 family chitosanase expressed in *E. coli* was suitable for the large-scale production of COS because 1 g of enzyme could hydrolyze up to 100 kg of chitosan [125]. A *B. subtilis* GH46 family chitosanase with fast reaction times has been expressed in *E. coli* and used to produce COS with a size range of DP1–DP6 starting with chitosan samples differing in DA [124,126]. The product distribution could be shifted to favor lower or higher DP by changing the type of chitosan substrate, the incubation temperature (30 °C, 50 °C) and the incubation time (2 min to 3 h) [124,126]. A unique chitinase D from *Serratia marcescens* (SmChiD) was found to display chitobiase activity on DP2 substrates and transglycosylation activity on substrates with a DP ≥ 3, resulting in products of DP1 and DP2 and the formation of oligomers of DP3–DP6 [133]. This enzyme is ideal for the production of longer-chain bioactive COS. Many chitinolytic and chitosanlytic enzymes have been characterized in natural extracts or produced as recombinant proteins for testing, revealing different reaction mechanisms and product properties. This provides a vast spectrum of different enzymes to generate products with a range of DP values, but the diversity and complexity of the enzymes makes it difficult to find the optimal candidate. In this regard, the enzyme yield achieved by natural or heterologous expression is a key factor which influences overall process productivity. Process development for the production of novel COS products must therefore balance the need for a specific reaction mechanism with the ability to produce sufficient quantities of each functional enzyme.

## 5. Fully Enzymatic COS Production

Many microbes can utilize chitin from marine or terrestrial organisms as a source of carbon and nitrogen [134,135,136]. Chitin is usually degraded extracellularly to monomers and dimers using mixtures of endo-chitinases, exo-chitinases and chitosanases. The simpler carbohydrates are then assimilated and metabolized. Despite these effective enzymatic mechanisms, a whole-bacterial downstream process that converts chitin to COS has not yet been achieved. This reflects the fact that exo-chitinases completely degrade chitin to yield monomers. Deleting or silencing these enzymes could potentially allow the production of COS but this would also prevent the bacteria utilizing chitin as a nutrient, therefore inhibiting their growth. The provision of alternative carbon sources such as glucose would allow bacterial growth but would suppress the expression of chitinolytic enzymes because bacteria tend to switch off unnecessary metabolic circuits if a preferred substrate is available. Therefore, whole-bacterial degradation processes are currently used solely for the commercial production of monomeric GlcNAc [137]. To overcome these limitations, in vitro degradation processes can be established using either naturally extracted or recombinant chitinolytic enzymes for the development of specific enzyme cocktails. To the best of our knowledge, a fully enzymatic process for the in vitro conversion of chitin into defined COS (i.e., enzymatic deacetylation and depolymerization steps using chitinases and/or chitosanases and CDAs) has not been reported. However, hybrid processes combining chemistry and biology have been established at the laboratory scale to generate defined COS. These semi-biological processes usually require chitin standard oligomers with a highly-defined DP and use CDAs alone or in combination to generate defined COS [48]. Alternatively, chitosan produced by chemical deacetylation can be depolymerized using chitinases and chitosanases to yield defined COS [120]. The environmentally friendly production of COS requires the establishment of fully enzymatic processes for the conversion of chitin to defined COS by combining chitinases and CDAs in a single process, thus avoiding the use of hazardous chemicals. However, due to the abundance of natural chitinolytic enzymes and their diverse substrate specificities and product characteristics, the underlying biocatalysis mechanism and process-relevant enzyme properties must be characterized first. Furthermore, for multi-step enzymatic reactions carried out using enzyme cocktails, the reaction conditions must be optimized for all enzymes in the process simultaneously. 

## 6. Design-of-experiments Approach for Multi-enzyme Process Optimization

**Figure 5:**
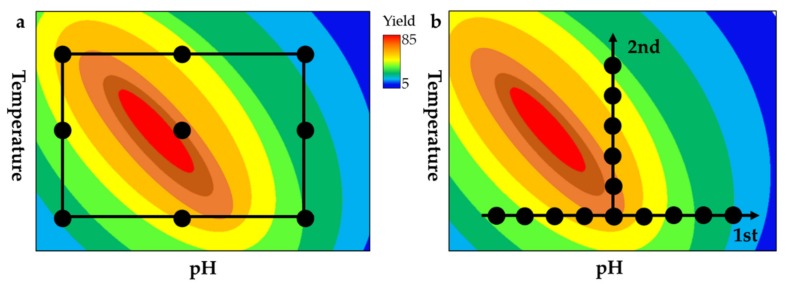
Comparison of a factorial design experiment (**a**) and the one-factor-at-a-time (OFAT) approach (**b**) for the maximization of the product yield by optimizing two representative process factors: pH and temperature. a: The factorial experimental design allows the investigation of all possible combinations of the two factors on different levels considering potential interdependencies between the factors within the design-space. Optimum conditions for both factors that give maximum yield can be determined. b: In the OFAT approach, both factors are investigated sequentially (1st, 2nd) neglecting factor interactions, leading to the loss of conclusive information and thereby missing the true maximum.

Standard process optimization involves the variation of one parameter while the others are held constant, which is known as the one-factor-at-a-time (OFAT) approach. This does not account for interactions between factors, so statistical experimental designs, also known as the design-of-experiments (DoE) approach, are more widely used for bioprocess optimization and were successfully applied to significantly improve biomass, protein and metabolite yields [138,139,140,141,142,143]. Statistical analysis can be used to simultaneously examine the effects of multiple input parameters on a given output, allowing conversion processes to be optimized in terms of product accumulation and any other relevant characteristics by considering how the input parameters interact [144,145]. As the OFAT does not take these factor interactions into account due to the sequential optimization of individual factors, putative beneficial effects are not assessed and often the true optimum Various specified designs are available for process optimization studies, including RSM for the quantitation of factor impacts on responses, mixture designs for the optimization of mixtures, and factorial designs to test screening factors (Figure 5) [146]. As mentioned above, statistical experimental designs have been successfully applied for the chemical degradation of chitin to chitosan and COS in order to assess factor interactions between the process variables reagent type and content, reaction time and process temperature [80,81]. For complex enzyme reactions, the product-related effects of various physical and biological factors can be investigated and quantified, including pH, temperature, reaction time, salt concentration, co-factors, enzyme combinations, and enzyme mixture ratios. Because DoE analysis reveals factor interactions, predictive models can be built that offer further information about process parameters to increase product yields and process efficiency [147,148]. Sequential rounds of DoE optimization can be carried out to optimize the overall process, reflecting the complexity and number of investigated factors. For example, initial factorial screening involves the investigation of multiple factors on different levels, revealing the significance of basic factor interactions. In the sequential approach, factors showing no (or insignificant) interdependencies can be excluded from the experiment and RSM is used to assess factor interdependencies in more detail [149]. The final predictive model can determine optimal factor combinations as well as predicted and extrapolated factor combinations to achieve a specific, desired output. Recently, a DoE approach was used to create optimized enzyme cocktails for the complete enzymatic hydrolysis of marine chitin to monomeric GlcNAc by testing 41 enzyme mixtures each comprising five different chitinases. A full cubic model and D-optimal design was used for optimization and similar saccharification yields (70–75%) and enzyme dosages to that of current lingocellulose processings could be achieved with the optimized cocktail [150]. A similar DoE approach has been described for the hydrolysis of lignocellulosic biomass. A quadratic design including five different enzyme mixture components was used for a two stage optimization process. Higher hydrolysis yields (% of glycan conversion) of the optimized cocktails (82.3 ± 4.4%) were observed compared to the two commercial cellulose cocktails Cellic^®^ CTec2 (70.0 ± 4.7%) and Cellic^®^ CTec3 (76.3 ± 0.8%) [147]. Synergistic minimal enzyme cocktails have also been developed for the saccharification of sugarcane biomass using a mixture model (simplex-centroid model) in which precise mixture ratios of three enzymes were compared at their reaction optima. As in the optimization study above, the optimized enzyme cocktail was compared to two commercial cellulose cocktails (Celluclast 1.5 L and Cellic CTect2) and comparable reducing sugar yields could be achieved [151]. For the conversion of chitin to COS, the DoE factors (enzymes, mix ratios, temperature, pH, salt, and cofactors) could be tested not only to increase overall product yields but also to achieve specific values for DP and DA.

In conclusion, it can be pointed out the large amount of different chitinolytic enzymes and variable physicochemical parameters allow for a far more open process development in terms of altered product properties compared to the chemical degradation process that is restricted by the limited amount of different process parameters.

Additional optimization approaches which are beyond the scope of this article include enzyme immobilization and directed enzyme evolution, each of which can also be used to improve productivity and alter product characteristics. Enzyme immobilization could be used to establish a continuous COS production system by immobilizing the enzymes on a matrix without disrupting their activity, and delivering the substrate continuously while the product is continuously removed. The space–time yields can therefore be increased and the costs simultaneously reduced due to the enzyme recycling, and product-dependent enzyme inhibition effects are avoided [152,153]. Directed evolution can be used to modify various properties including enzyme activity, temperature/pH stability, substrate specificity, and reaction mechanisms. This can be achieved by random mutagenesis followed by library screening using a method that selects for enzymes with improved properties. Alternatively, if the crystal structure is known, amino acids occupying key positions in and around the catalytic site or substrate-binding site can be systematically exchanged by site-directed mutagenesis followed by direct comparisons to determine activity and specificity [154,155,156]. However, even with these approaches, process optimization must still take into account multiple interacting factors, and statistical experimental designs will remain a valuable strategy to maximize productivity and tailor the properties of enzymes to achieve the production of specific, defined COS. 

## 7. Conclusion and Future Prospects

In this review, we have discussed the characteristics and applications of chitosan and COS, highlighting the structure–function relationship in terms of DP, DA and PA. Controllable degradation processes are required to generate consistent COS products suitable for specific downstream applications. The optimization of chemical chitin degradation can be achieved by combining different catalysts and by modifying the physicochemical parameters to produce COS in a controllable manner, but it is not yet possible to produce individual species of COS with the DP, DA and PA fully defined. Furthermore, all such processes involve the use of environmentally hazardous chemicals. A synthetic biology approach has recently been applied in *E. coli* to achieve the biosynthesis of defined COS starting from the substrate glucose by expressing the *nodC* and *nodB* genes encoding chitin synthase and chitin deacetylase, respectively [34]. This in vivo approach is interesting from a metabolic engineering perspective and can generate defined COS for functional analysis, but the processing of biowaste might be more sustainable, especially in terms of large-scale production. Chitin from crustacean shells is one of the most abundant biological waste materials in the world, and its valorization by conversion to COS by whole-bacterial or in vitro degradation is therefore preferable to *de novo* COS biosynthesis. Biological in vitro approaches using chitinolytic enzymes are more attractive compared also to chemical treatments because they involve mild reaction conditions, the enzymatic degradation process is more controllable due to the selectivity of different chitinases/chitosanases and CDAs, and the absence of hazardous chemicals eliminates the risk of carryover into products intended for medical applications. A complete in vitro enzymatic degradation processes from chitin to defined COS has yet to be established due to the lack of suitable enzymes and a poor understanding of the complex nature of multi-stage enzymatic reactions. Current research is therefore focusing on the isolation and characterization of novel chitinolytic enzymes to determine their mechanisms of action. However, these studies tend to use pre-treated materials such as pure chitosan or colloidal chitin, and further research is needed to achieve the direct conversion of biomass-derived insoluble chitin into COS. Enzymatic in vitro approaches are more expensive than chemical degradation due to the high costs of enzyme production and purification. To address this issue, DoE might help to maximize the cost–benefit ratio for enzyme and COS production. Furthermore, alternative conversion methods can be developed to reduce costs further. Synthetic biology and metabolic engineering could be used to integrate the enzymes needed for COS production in one cell, allowing the whole-bacterial limited degradation of chitin while eliminating the costs of enzyme isolation and purification. Furthermore, the discovery of unique reaction mechanisms will allow the production of novel and precisely-defined COS, making enzymatic conversion much more competitive with chemical processes and therefore more market ready. Current political and socio-economic trends reveal the increasing social demand for sustainable and environmentally friendly processes for the generation of energy and biobased products. An enzymatic conversion process that can match or even exceed the results of chemical degradation has the potential to co-exist as an industrial process for the production of defined COS. Such enzymatic processes are likely to succeed initially in the niche market for therapeutic COS products, where physiological activity and consistent product quality and safety are more important than production costs. Algal, fungal and insect chitin will probably attract increasing attention because it is easier to isolate chitin from these sources than crustacean waste, resulting in a more consistent starting material that will improve the quality of the final COS product. Even so, crustacean biomass is likely to remain the primary source of industrial chitin because it is an abundant and inexpensive waste stream that (unlike algae, fungi and insects) does not have to be cultured or specially bred. Finally, we discussed the use of DoE methods to experimentally assess factors that have a significant effect on the enzymatic degradation of chitin and the characteristics of COS. This approach may allow the development of enzyme cocktails that achieve the more efficient enzymatic production of defined COS including entirely new COS with unique bioactive properties.

## Figures and Tables

**Table 1:** Isolated or recombinant fungal (above the dotted line) and bacterial (below the dotted line) chitin deacetylases (CDAs) that convert oligomeric chitin substrates to partially deacetylated chitosan oligomers (COS) with various patterns of acetylation. Abbreviations: n.r. = not reported; DP = degree of polymerization; A = acetylated monomer; D = deacetylated monomer; index n-m = terminal distance and locus where CDA converts A to D.

Enzyme	Source Organism	Expression Host	Substrate	COS Product	Literature
ClCDA	*Colletotrichum* *lindemuthianum*	Natural isolate	DP2–DP6	D_n_	[97]
MrCDA	*Mucor rouxii*	Natural isolate	DP3	D_n_	[95]
ScCDA1	*Saccharomyces cerevisiae*	*S. cerevisiae,* *Pichia pastoris*	DP2–DP6	n.r.	[104]
ScCDA2	*S. cerevisiae*	*S. cerevisiae,* *Pichia pastoris,* *E.coli*	DP2–DP7	n.r.	[105,106]
FvCDA	*Flammulina velutipes*	*Pichia pastoris*	DP2–DP6	n.r.	[107]
RcCDA	*Rhizopus circinans*	*Pichia pastoris*	DP6	n.r.	[108]
PaCDA	*Podospora anserinas*	*Hansenula polymorpha*	≥DP2	D_n_	[109]
AnCDA	*Aspergillus nidulans*	*E.coli*	DP2–DP5	D_n_	[110] [114]
PgtCDA	*Puccinia graminis*	*E.coli*	DP4-DP6	AAD_n-2_	[103]
PesCDA	*Pestolotiopsis* sp.	*E.coli*	DP4-DP6	AAD_n-3_A	[111]
PcCDA	*Pochonia chlamydosporia*	*E.coli*	DP4, DP5	ADDA_n-3_	[94]
SwCOD	*Shewanella woodyi*	*E.coli*	DP2–DP4	AD; [ADA_n−2_]	[100]
SbCOD	*Shewanella baltica*	*E.coli*	DP2–DP4	AD; [ADA_n−2_]	[99]
ArCE4A	*Arthrobacter* sp.	*E.coli*	DP2–DP6	D_n−1_ A	[102]
NodB	*Rhizobium* spp.	*E.coli*	DP1–DP6	ADA_n-2_	[48]
VcCOD	*Vibrio chloreae*	*E.coli*	DP2–DP6	ADA_n-2_	[110]

**Table 2:** Isolated or recombinant fungal (above the dotted line) and bacterial (below the dotted line) chitinolytic glycosyl hydrolases used for the conversion of chitinous substrates to chitosan oligomers (COS) with different degrees of polymerization (DP). DA = degree of acetylation. GlcNAc = *N*-acetylglucosamine.

Enzyme	Source Organism	Expression Host	Substrate	COS Product	Literature
Chitinosanase	*Alternaria alternata*	Natural isolate	Chitosan DA 40–70%	Cleavage after GlcN-GlcNAc	[119]
Chitosanase	*Purpureocillium lilacinum* CFRNT12	Natural isolate	Colloidal and crystalline chitosan	DP 2–6	[120]
Chitinase Chi1	*Myceliophthora thermophila* C1	*Myceliophthora thermophila* C1	Chitosan Mw (100, 600, and 3000 kDa); DA (77, 78, 88, 90%)	DP 2–12	[121]
Chitosanase	*B. subtilis*	*B. subtilis* PT5	α and β type chitosan	DP 2–4	[122]
Chitinase-D	*Serratia proteamaculans*	*Escherichia coli*	Chitosan DA 35% and 61%	DP 2–12	[123]
Chitinase-D	*Serratia marcescens* GPS5	*E. coli*	Colloidal chitin, chitosan DA 10%	DP 1–8	[124]
GH46 family chitosanase	*Bacillus subtilis* (BsCsn46A)	*E. coli*	Chitosan DA 15, 30 and 60%	DP 2–15	[125,126]
GH46 family chitosanase	*Gynuella sunshinyii*	*E. coli*	Chitosan DA 95% COS DP 2–6	DP 2–7	[127]
GH8 family chitosanase	*Bacillus strain*	*E. coli*	Chitosan DA > 90%	DP 5.5 (mean)	[114]
